# A data-driven model for early prediction of need for invasive mechanical ventilation in pediatric intensive care unit patients

**DOI:** 10.1371/journal.pone.0289763

**Published:** 2023-08-04

**Authors:** Sanjukta N. Bose, Andrew Defante, Joseph L. Greenstein, Gabriel G. Haddad, Julie Ryu, Raimond L. Winslow

**Affiliations:** 1 Enterprise Data and Analytics, University of Maryland Medical System, Linthicum Heights, MD, United States of America; 2 Rady Children’s Hospital, San Diego, CA, United States of America; 3 Institute for Computational Medicine, Johns Hopkins University, Baltimore, MD, United States of America; 4 Division of Respiratory Medicine, Department of Pediatrics, University of California San Diego, La Jolla, CA, United States of America; 5 Department of Neurosciences, University of California San Diego, La Jolla, CA, United States of America; 6 Roux Institute at Northeastern University, Portland, ME, United States of America; 7 Department of Bioengineering, Northeastern University, Boston, MA, United States of America; First Technical University, NIGERIA

## Abstract

**Rationale:**

Acute respiratory failure is a life-threatening clinical outcome in critically ill pediatric patients. In severe cases, patients can require mechanical ventilation (MV) for survival. Early recognition of these patients can potentially help clinicians alter the clinical course and lead to improved outcomes.

**Objectives:**

To build a data-driven model for early prediction of the need for mechanical ventilation in pediatric intensive care unit (PICU) patients.

**Methods:**

The study consists of a single-center retrospective observational study on a cohort of 13,651 PICU patients admitted between 1/01/2010 and 5/15/2018 with a prevalence of 8.06% for MV due to respiratory failure. XGBoost (extreme gradient boosting) and a convolutional neural network (CNN) using medication history were used to develop a prediction model that could yield a time-varying "risk-score"—a continuous probability of whether a patient will receive MV—and an ideal global threshold was calculated from the receiver operating characteristics (ROC) curve. The early prediction point (EPP) was the first time the risk-score surpassed the optimal threshold, and the interval between the EPP and the start of the MV was the early warning period (EWT). Spectral clustering identified patient groups based on risk-score trajectories after EPP.

**Results:**

A clinical and medication history-based model achieved a 0.89 area under the ROC curve (AUROC), 0.6 sensitivity, 0.95 specificity, 0.55 positive predictive value (PPV), and 0.95 negative predictive value (NPV). Early warning time (EWT) median [inter-quartile range] of this model was 9.9[4.2–69.2] hours. Clustering risk-score trajectories within a six-hour window after the early prediction point (EPP) established three patient groups, with the highest risk group’s PPV being 0.92.

**Conclusions:**

This study uses a unique method to extract and apply medication history information, such as time-varying variables, to identify patients who may need mechanical ventilation for respiratory failure and provide an early warning period to avert it.

## Introduction

Acute respiratory failure (ARF) in children is a leading cause of morbidity and mortality [1–3]. Respiratory failure occurs when the body is unable to get enough oxygen into the blood from the lungs or remove enough carbon dioxide from the blood. Acute respiratory failure is one of the top reasons children are admitted to an intensive care unit [4]. Infants and young children are at a higher risk of developing acute respiratory failure than adults for various reasons, such as different mechanical, muscular properties, and lower reserves in their respiratory system than adults. ARF can occur in children like in adults because of infections, asthma attacks, neuromuscular conditions, congenital heart disease, airway obstruction, trauma, or are drug related [2, 3, 5]. Pediatric acute respiratory failure symptoms include difficulty breathing, rapid breathing, bluish skin, lips, and fingernails (known as cyanosis), and confusion [6]. Immediate treatment includes giving oxygen until the underlying cause is identified, and it usually involves a gradual increase in respiratory support such as non-invasive ventilation and ultimately intubation with mechanical ventilation. Asthma and airway reactivity are very common causes of ARF in children [1, 7]. For this condition, it is generally recommended to avoid mechanical ventilation and treat it with non-invasive and other forms of respiratory support. In addition, supplemental oxygen and non-invasive ventilation are adequate for many children who have acute respiratory failure, but mechanical ventilation becomes necessary in severe cases [8].

Invasive mechanical ventilation (MV) is used as a life-saving intervention for ARF, but this carries a high risk of lung injury and intubation-associated infection [9]. More than half of patients with ARF required MV [3]. A prior meta-analysis of twenty-nine studies reported an incidence rate of 2.3% per 100,000 patient years and a mortality rate of 34% for pediatric acute respiratory distress syndrome [6, 10]. Mechanical ventilation is an invasive process that involves intubation with an artificial airway to connect to a ventilator [11]. Since a conscious patient typically cannot tolerate intubation, mechanical ventilation necessitates sedation and paralysis, which impairs the patient’s ability to interact or communicate. Alternations include infective airway clearance, increased risk of nosocomial infections, the need for enteral feeds via a tube or intravenous fluids, and general muscle wasting due to prolonged paralysis. A sufficient early warning of a high risk of MV could help prescribe medical interventions to potentially avoid the need for MV.

Unlike acute respiratory distress syndrome (ARDS) in adults, there are very few studies focused on predicting ARF and the need for MV in children. The literature on MV prediction is limited to specific patient populations requiring prolonged MV [12–16]. There are no existing studies that have been successful in predicting the need for MV in a general PICU population. Several scoring systems [17] that are based on specific physiologic or laboratory data are used most frequently in intensive care units to predict general risk of organ failure, such as the Sequential Organ Failure Assessment (SOFA) [18, 19] score or mortality, such as the Acute Physiologic Assessment and Chronic Health Evaluation (APACHE) [20] score. The incorporation of data from the Internet of Things (IoT) [21, 22], a network of physical objects with embedded sensors and actuators that can collect and exchange data, can also be advantageous for these scoring systems. The IoT can offer real-time analytics, automation, and monitoring for the equipment and inventory as well as for the productivity and efficiency of staff and patients. However, none of these scoring systems have been targeted to respiratory failure in pediatric patients [23].The Pediatric Early Warning Score (PEWS) [24] is a frequently used scoring system in pediatric inpatient medical units to identify patients at risk for patient deterioration, in contrast to many other scoring systems adapted for children. Although the PEWS scoring system was designed for use on the medical floor, researchers have also applied it to intermediate medical units. Since there are no published early warning prediction models for children who need mechanical ventilation, the PEWS scoring system was investigated using this dataset.

This study presents a novel approach for combining static and dynamic variables extracted from electronic health records, including medication history, to create a prediction model for identifying patients who are likely to receive MV due to ARF.

## Methods

### Data description

**Fig 1:**
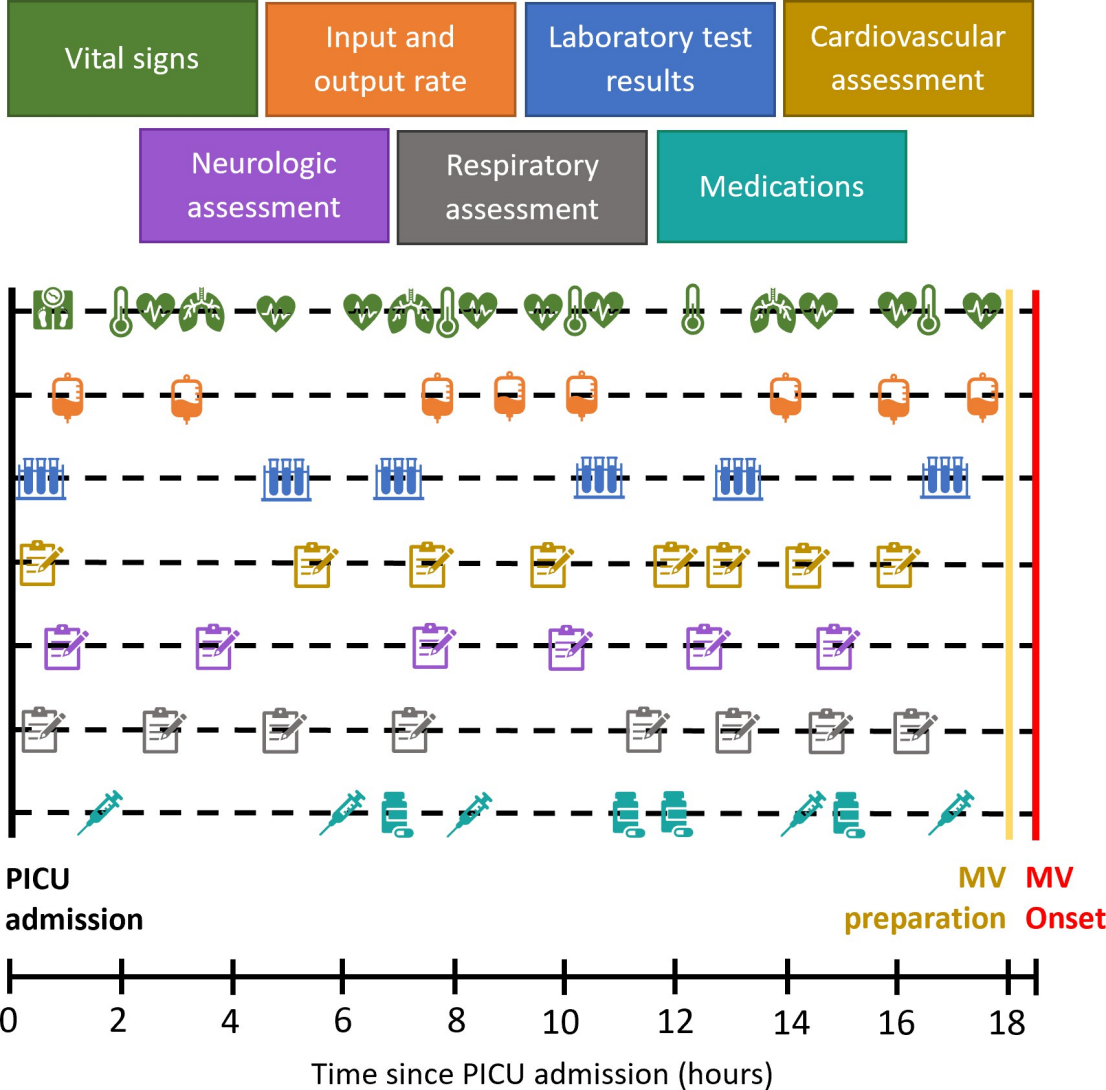
Dataset feature description. Each of the above feature labels can represent multiple measurements; for example, vital signs can represent heart rate, respiratory rate, oxygen saturation, blood pressure etc. The orange box on the input and output rate features indicates that the rate of inputs and outputs were calculated in a continuous manner using the preceding 6 hours.

**Fig 2:**
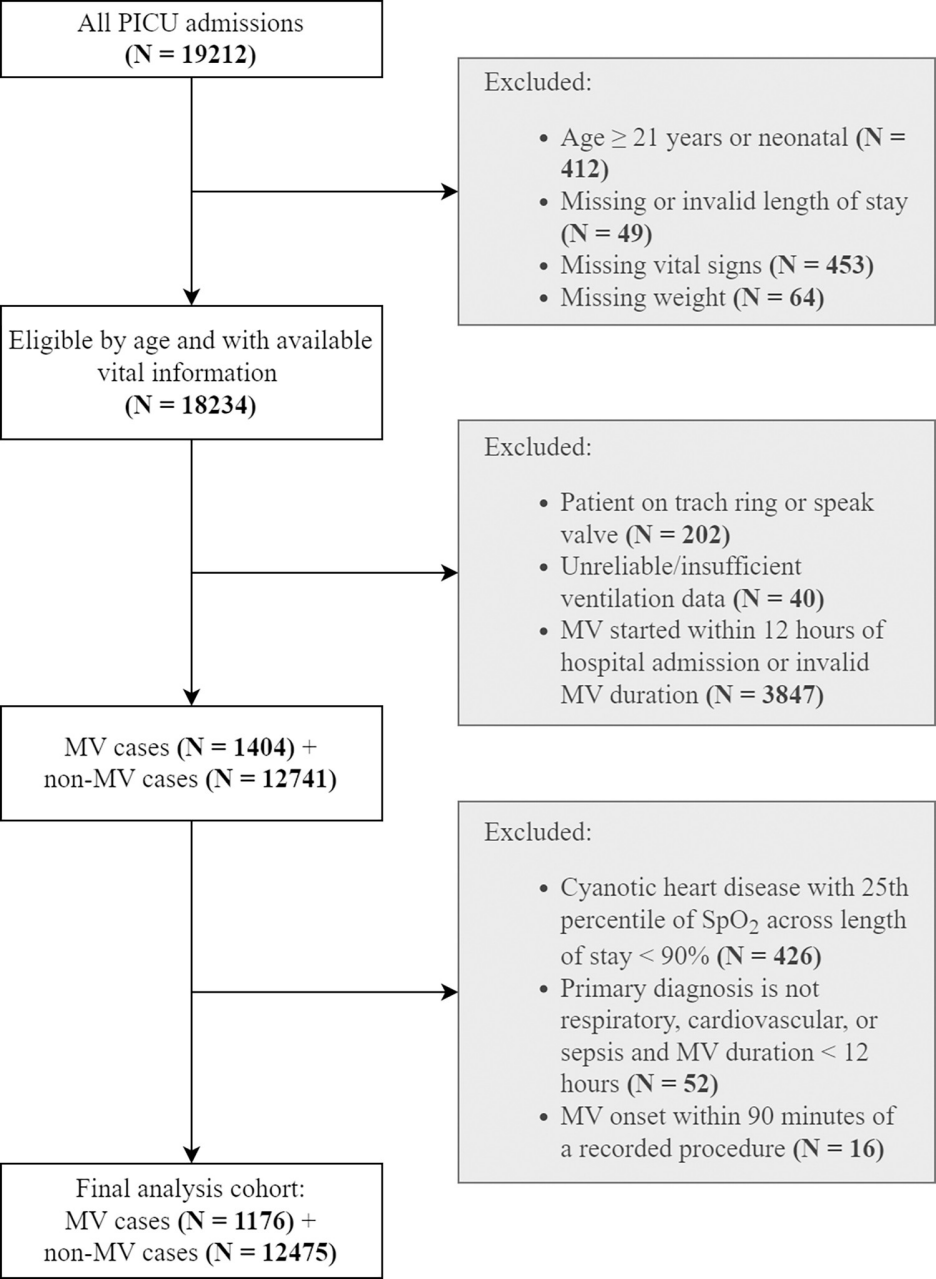
Flowchart of study inclusion-exclusion criteria.

This retrospective, single-center study utilized electronic health record (EHR) data from patients admitted to the Pediatric Intensive Care Unit (PICU) at Rady’s Children’s Hospital in San Diego, California between 1/01/2010 and 5/15/2018. IRB approval was obtained from Rady/UCSD. Only the research team had access to clinical data records to validate data for accuracy, once data was validated, only coded identifiers were used in the dataset. All patients aged 0 to 21 years with non-missing age, gender, weight, PICU length of stay (LOS), and at least one recorded vital sign were included. This model was created using routine structured nursing and respiratory therapy assessments, vital signs, medication administration records, respiratory support documentation, and labs (Fig 1). To make the prediction model more applicable, patients with tracheostomy or cyanotic heart disease whose baseline oxygen saturation was less than 90% for at least 75% of the hospital stay were excluded. Also excluded were records in which the first ventilator event was likely the result of a transcription error (manually verified) or insufficient ventilation information. Patients who received MV within 12 hours of PICU admission were excluded because there was insufficient data prior to the onset of MV. To rule out non-respiratory failure MV, such as elective procedures, MV patients were excluded whose discharge diagnosis did not include at least one respiratory or cardiovascular diagnosis, or sepsis, or MV duration was less than 12 hours, or MV onset occurred within 90 minutes of a documented medical procedure/surgery. The absence of ventilation flowsheet information indicated that patients were receiving only room air and did not need respiratory support (Fig 2).

### Feature preprocessing for non-medication features

Since all the data extracted from electronic health records were recorded at non-uniform time intervals, all features were resampled at every 5-minute intervals and carried forward until the next observation. Categorical features were converted to one-hot encoded vectors, which implies that if a categorical variable had N-discrete values, then each of those values were represented by N different binary variables. Numerical variables which vary with patient characteristics such as age, height, weight, and gender, were standardized by converting to z-scores ($z=\frac{x-\mu }{\sigma }$, *z*: z-score, *x*: raw value of variable, *μ, σ*: mean and standard deviation) using the Harriet Lane Handbook of Pediatrics’ range of normal values for individual physiologic variables [24]. Height and weight features were then converted to z-scores and missing values of height were replaced with z-score = 0. Even after carrying forward observations, there were still some missing data, and some measurements, such as invasive lab tests, were not done often on patients who were relatively stable. For an XGBoost model [25, 26], missing values were not required to be imputed, but for the lasso-GLM model [27] model, missing values were replaced with the median of the observed data for each variable across the training dataset.

### Model based feature selection

One of the primary reasons for selecting XGBoost and lasso-GLM was that both can handle a large number of features and automatically select a subset of features that yields the best classifier. Lasso-GLM (equivalent to a regularized logistic regression) introduces sparsity to the set of features in the model through the addition of L1-norm (number of non-zero values) of model coefficients to the objective function of a generalized linear model. XGBoost is a non-linear ensemble decision tree-based method that sequentially grows decision trees with a specified maximum depth and minimum weight for each child node. In each iteration, a new decision tree is constructed using a subset of training data and a random subset of features, with the goal of minimizing the sum of squared error between the known outputs and the expected model outputs based on previous iterations. The two parameters, maximum depth of each decision tree and the fraction of features to subsample at each iteration, constrain the XGBoost model to choose an even smaller subset from the randomly selected subset at each iteration. XGBoost is known to be more resistant to model overfitting and feature multicollinearity. As a result, while both XGBoost and lasso-GLM can automatically select the most relevant features that would result in the best prediction model, they are more likely to select different features due to differences in their underlying methodology, which is especially noticeable when features are highly correlated. The only disadvantage is that while the features chosen by these models are predictive, clinical causality may be confounded by other related variables.

The final set of features used to develop and test the prediction models consisted of 188 features derived from respiratory, cardiovascular, and neurologic nursing assessments, laboratory test (blood gases, blood chemistry) flowsheets, as well as additional features from medication records (Fig 1). Description of feature selection and processing are provided in S1 Fig 1, and S1-1, S1-2 Tables in the S1 Appendix.

### Medication based features

Two different strategies were used to harness medication-based features. The first strategy used a standard method of using continuous-time binary indicators for each medication to indicate whether or not it had been administered over the previous 6-hour period. Because several periodically or continuously administered medications in the dataset were typically prescribed in 4–6 hour intervals, 6-hour time windows were chosen. In order to use this method, a subset of drugs were chosen based on whether those were given to at least 10% of MV patients or whose prevalence for at least one dose per patient differed by more than 5% points between the MV and no-MV group (see S1 Table 1 in S1 Appendix for the list selected medications).

**Fig 3:**
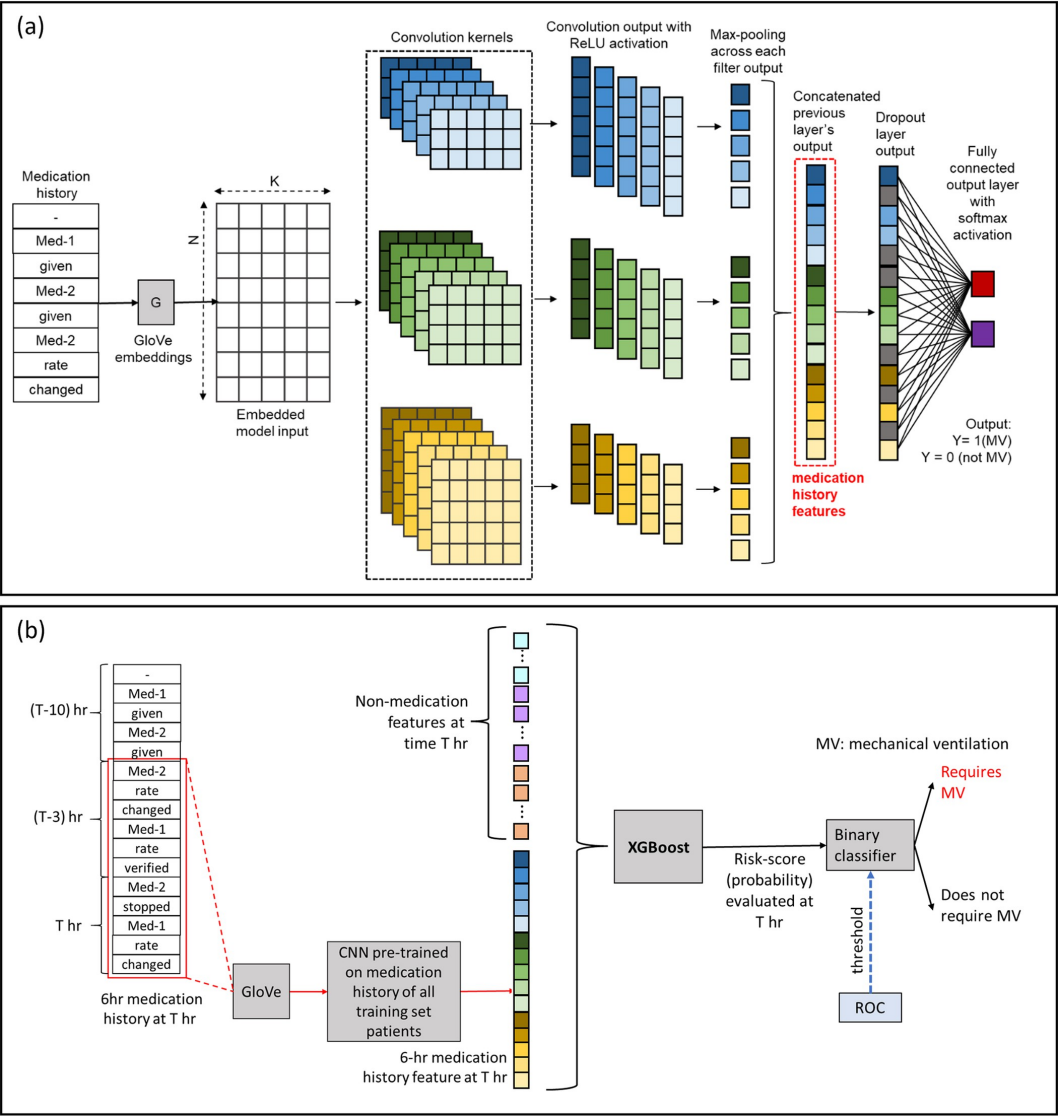
Medication history-based feature computation model. (a) Medication history-based feature computation model (b) Use of medication history features in the overall prediction model along with non-medication features.

The second method for incorporating medication data not only relied on the medications given at the same time instant but also the history of other medications given to each patient with respect to the current time. To harness the medication history as features in the prediction model, a novel application of a convolutional neural network (CNN) model was proposed based on the network architecture introduced by Kim et al. [28, 29] to perform sentence classification for natural language processing applications. Fig 3 shows the architecture of the model used in the study, which was very similar to the architecture proposed by Kim et al. [28] with different number and sizes of convolutional filters. This model uses a fixed embedding layer, followed by a single convolutional layer consisting of five 1-dimensional convolutional filters each of dimension 4x20, 5x20, and 6x20, followed by max-pooling and then a drop-out layer for regularization (prevents overfitting) and finally a soft-max layer, which produces a binary output. The number of convolutional filters and the filter dimensions were chosen based on optimal model performance obtained by training models with different combination of number and sizes of filters treated as tunable hyperparameters. The embedding layer in this model takes in the medication history within the 6-hour period prior to a given time point in the form of a sequence of medication names and actions such as “Medication-1 given medication-2 given medication-1 rate changed medication-2 stopped” and maps this structured sentence to a numerical vector, for example [0.01, 0, 0.5, 0.004, 0.6, 0.78, 0, 0.1, 0, 0]. There were a total of 1436 unique medications found in the training set and the corresponding unique actions used for these medications were “given”, “rate changed” and “stopped”. This allows the model to represent words as numeric values that can be used as inputs by the convolutional layer which trains itself based on the medication history provided and corresponding classification output provided as training data to the CNN model. The embedding layer used in the CNN model is a fixed non-trainable layer, which used GloVe (global vector embedding) [30] to represent individual words as numerical vectors and therefore, a sentence was simply constructed by concatenating the numeric representation for each word. The GloVe implementation from the R package text2vec [31] was utilized. GloVe is an unsupervised representation learning technique that uses the co-occurrence frequency for a pair of words which usually occur within a specified distance from each other in a sentence (known as the context window size). The dimension of word embedding produced by GloVe and the context window length are variable parameters to be determined by user based on downstream tasks. Therefore, GloVe essentially serves as a dictionary of words that is trained on a fixed corpus and is later used to map words in its dictionary to numeric features of length specified by the embedding dimension. The CNN model was trained with different values of embedding dimension and context window length provided to GloVe and found that the model’s performance was relatively less variable with respect to these parameters. The final set of parameters determined from hyperparameter tuning were a context window size of 25 words and an embedding dimension of 10. Finally, the entire history of medication up to the time of MV preparation for each target (MV) case and the medication history during the entire length of stay (LOS) for each control (no-MV) case were used as inputs and the binary outcome MV = 1, no-MV = 0 was used to train the CNN. The CNN model thus obtained after 10-fold cross validation on the training set, was used as a pre-trained model and the layer just before the binary output was used as learnt representation of the medication history that could be used to derive multidimensional medication history features along with non-medication features for building the overall prediction model using XGBoost. For generating medication history features at a given time, the sequence of medications given, rate changed and stopped in the previous 6-hour window were collated and passed through the trained CNN to obtain the medication history features a shown in Fig 3. Fig 3(B) shows how the medication history feature was used in conjunction with other features at any given time.

### Model building and evaluation

A set of four different models were trained using different set of input features:

- Model-A: It contains non-medication features and medication history features derived from the pre-trained CNN described in the previous section.
- Model-B: It contains non-medication features and medication indicator features for medication listed in S1 Table 1 in S1 Appendix.
- Model-C: It contains only non-medication features
- Model-D: It contains only medication history features (included in Model-A).

The performance of the above models was compared and the performance of the two best performing models among these were also compared by using two different machine learning methods: XGBoost (extreme gradient boosting [25, 26] and lasso-GLM [27].

XGBoost was the default method for building prediction models to identify increased risk for MV in PICU patients. A 30 minute time window prior to MV onset was labelled as the MV preparation time during which hypnotics and anesthetics were routinely administered in preparation for intubation and subsequent MV initiation. To avoid misidentification of physiologic response to preparation for MV as a predictor, data collected between MV preparation and MV onset times were excluded during training and testing phases.

The entire analysis was performed using R version 3.6.3 [32] and the R packages xgboost version 1.0.0.2 [33] and glmnet version 3.0.2 [34] were used to implement XGBoost and lasso-GLM models respectively. The R packages text2vec version 0.6 [35] was used to convert text to numeric vectors and keras version 2.3.0.0.9000 [36] to train the CNN for extracting medication history features.

Our training set was created by randomly selecting 80% of the patients stratified by MV outcome, gender, age group, and non-invasive positive pressure ventilation status, and the remaining 20% were held out as test set at the onset of the study. All samples in the 12-hour period preceding MV preparation from MV cases and an equal number of randomly selected samples from no-MV cases from the training set were used to train the model. The model was then cross-validated on the entire PICU course until MV preparation time for the MV group and the entire hospital course for the no-MV group within the training set. During model training, XGBoost creates a binary classifier that can distinguish samples from positive and negative classes. The corresponding model output is the probability of the sample belonging to the positive class given the features observed at a time point. This time-varying probability has been defined as the “risk-score” throughout this study. The optimal threshold risk score was obtained from the receiver operating characteristic (ROC) based on maximum F1 score [37].If the risk-score of the patient exceeded the threshold at any time between time of PICU admission and MV preparation time, the binary classifier generated a positive prediction for MV and a negative prediction if the risk-score of a patient never exceeded the threshold during their LOS. The first instance where the risk-score crossed the threshold was defined as the *early prediction point (EPP)* and the duration between EPP and MV onset was defined as the *early warning time* (EWT).

### Performance metrics

The performance metrics reported in the study to compare models were

- AUROC: Area under the receiver operating characteristic (ROC) curve
- AUPRC: Area under the precision-recall (PR) curve
- Sensitivity: Also known as recall or true positive rate. It is the ratio of true positives to the number of observed positives.
- Specificity: Also known as true negative rate. It is the ratio of true negatives to the number of observed negatives.
- Positive predictive value (PPV): Also known as precision. It is the ratio of true positives to the number of predicted positives.
- Negative predictive value (NPV): It is the ratio of true negatives to the number of predicted negatives.
- F1 score: It is defined as $\frac{2\times sensitivity\times PPV}{sensitivity+PPV}$. F1 score is often used as a criterion for threshold selection from ROC for datasets with high class imbalance.
- Early warning time (EWT): Lead time between the time of early prediction and MV onset.

### Statistical hypothesis tests

Wilcoxson rank-sum test and Kruskal-Wallis test were used for testing the association of continuous variables across categorical factors with 2 levels and more than 2 levels respectively. For categorical variables, Fisher exact test was used to determine association with other categorical factors. Cochran-Armitage test was used to test the presence of linear trends in binary variables across ordinal factors.

### Risk grouping using spectral clustering

Spectral clustering [38] is an efficient, graph theory-based clustering technique that can identify clustering patterns in high-dimensional data by computing the eigenvalues of the adjacency matrix (also known as the similarity matrix) between each pair of data points. The distance metric used to construct the similarity or adjacency matrix and the criteria used to determine the number of clusters vary in scientific literature, but the overarching mathematical underpinning remains the same. Our analysis used a KNN (K-nearest neighbor) based adjacency matrix and used the maximum eigengap criteria to determine number of clusters as described by von Luxburg [38].

Since spectral clustering is an unsupervised clustering method, it was applied to the risk-score trajectories within 0 to 6 hours from the time of early prediction within the training set to generate different risk-groups based on the clustering labels. The stability of these clusters was assessed on the training set by repeated subsampling of the training set without replacement (bootstrapping) and computing the maximum Jaccard index between the cluster assignments on each subset and the labels obtained on the entire training sample. The final parameters obtained for model-A was K = 200 which yielded three distinct clusters with an average Jaccard index > 0.9 from the stability analysis. The test set samples were finally assigned new risk-group labels with the help of KNN using the training set cluster labels as reference. Similar clustering approach has been applied in prior studies on prediction of multiple organ dysfunction [39] and septic shock [40] in pediatric population.

### Feature importance

Two different feature importance metrics were reported in this study, including XGBoost’s built-in model-based feature importance metric “gain” and “SHAP value”, a model-agnostic metric for feature contribution or importance. These two are inherently very differently computed and offer different perspectives on feature importance in a non-linear model like XGBoost. While the gain metric provides an insight into which features had the highest mean reduction in Gini impurity during the decision tree building process, it is not consistent and non-comparable with other methods. On the other hand, SHAP values [41, 42] are a model-agnostic consistent metric that can provide a quantitative measure of the expected value of difference in the predicted probability when a certain feature is present or missing given all other features are observed. The global SHAP feature importance can be evaluated by averaging the magnitude of SHAP values across all training samples for each feature. The most significant advantage of SHAP is the local explanation of each prediction on new data used for testing, as opposed to global feature importance metrics, which are computed solely on observed training data. The absolute values of SHAP reflect the magnitude of the association with the target outcome, and the sign indicates whether the association increases or decreases the odds of the outcome. The average absolute SHAP values computed across training data is reported as a general global feature importance metric.

## Results

### Study population characteristics

The retrospective dataset in this study had 14,192 PICU encounters. The final study population after applying the inclusion criteria outlined in Fig 2 included 13651 PICU encounters (1176 received MV, 12475 did not, 8.6% MV prevalence). The baseline characteristics of the included study population are shown in Table 1. The median age across MV cases was 1.2 years, compared to 4.8 years (p-value <0.001 using Wilcoxson ranksum test) among those who did not receive MV (no-MV). The median PICU LOS in MV cases was more than 6 times higher than in no-MV cases (19.4 vs 3.1 hours, p-value <0.001 using Wilcoxson ranksum test). The proportion of cases with non-invasive ventilation (NIV) prior to MV within MV cases was higher than the proportion of NIV in no-MV cases (28.4% vs 10.7%, p-value <0.001 using Fisher exact test). The distribution of gender, race, and ethnicity were similar in MV and no-MV groups.

**Table 1:** Study population characteristics.

Characteristics	All included PICU encounters (N = 13651)	Received mechanical ventilation?		p-value
		True	False	
		(N = 1176)	(N = 12475)	
Age* (years)	4.5 [1–12.2]	1.2 [0.2–9]	4.8 [1.1–12.4]	<0.001
Length of stay* (days)	3.6 [1.7–7.8]	19.4 [11.7–37.1]	3.1 [1.6–6.2]	<0.001
Gender^†^				
Male	7701 (56.4%)	627 (53.3%)	7074 (56.7%)	0.027
Female	5950 (43.6%)	549 (46.7%)	5401 (43.3%)	0.027
Race^†^				
White	6454 (47.3%)	577 (49.1%)	5877 (47.1%)	0.2
Black	859 (6.3%)	60 (5.1%)	799 (6.4%)	0.079
Asian	713 (5.2%)	76 (6.5%)	637 (5.1%)	0.054
Pacific Islander	194 (1.4%)	34 (2.9%)	160 (1.3%)	<0.001
Other	5431 (39.8%)	429 (36.5%)	5002 (40.1%)	0.016
Ethnicity^†^				
Hispanic	6558 (48%)	533 (45.3%)	6025 (48.3%)	0.054
Non-Hispanic	7060 (51.7%)	643 (54.7%)	6417 (51.4%)	0.035
Other	33 (0.2%)	0 (0%)	33 (0.3%)	0.111
Non-invasive positive pressure ventilation (NIV)^†^ (prior to MV or anytime for non-MV cases)	1664 (12.2%)	334 (28.4%)	1330 (10.7%)	<0.001

* Expressed as median [IQR] and p-values for these variables were calculated using Wilcoxson ranksum test.

^†^ Expressed as N (prevalence in %) and p-values for these variables were calculated using Fisher exact test.

### Prediction model performance and selection

**Fig 4:**
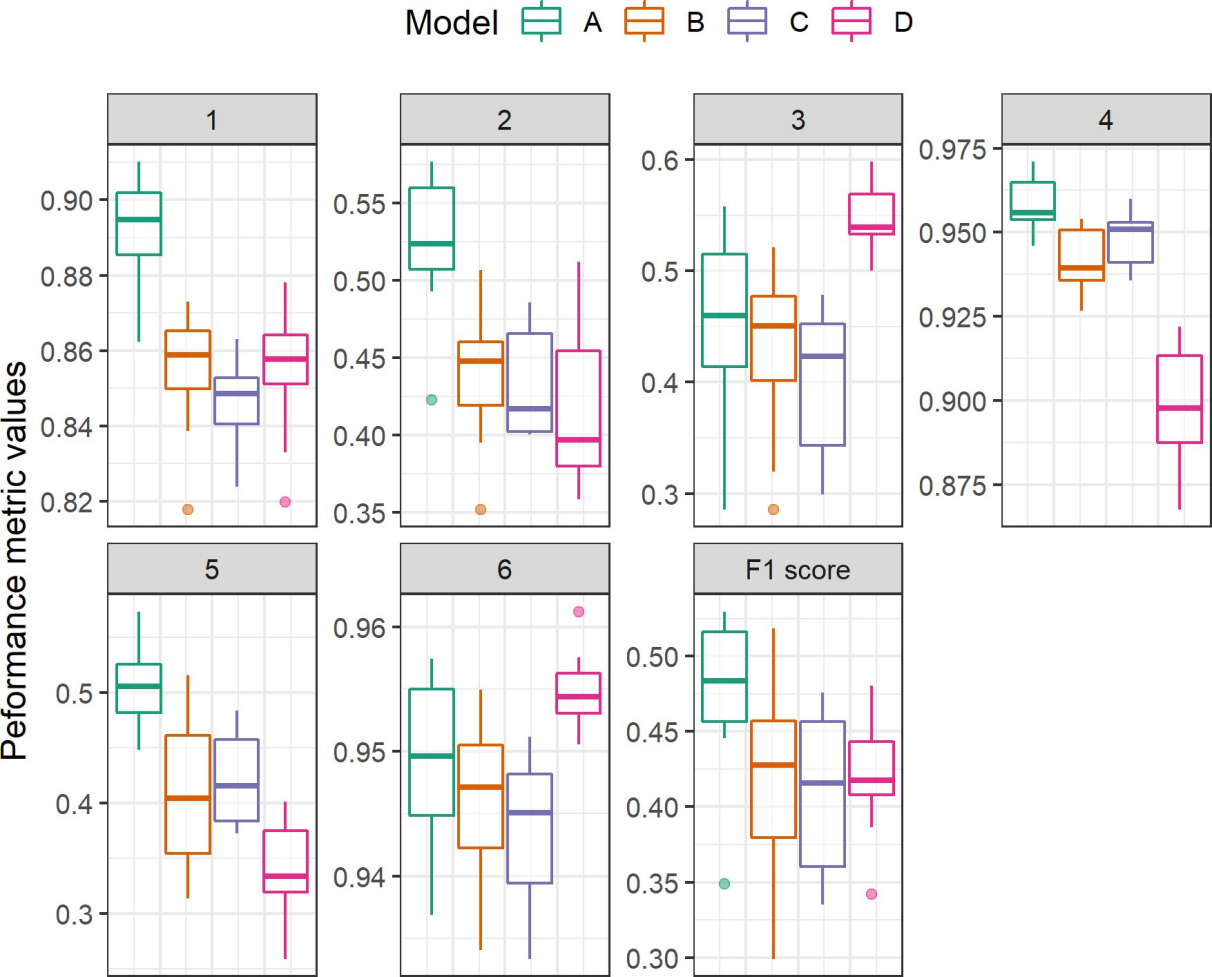
Performance metrics from 10-fold cross validation on training set using the tuned set of hyperparameters for XGBoost.

Models were created to test two different methods of incorporating medication data in addition to the clinical features. Model A used a combination of non-medication features (vitals, lab results, input output etc.) along with medication history features derived from a pretrained CNN. The CNN uses natural language processing to identify medication features such as “change in dose” and medication grouping combinations rather assessing a single medication independently (Fig 3). Model B utilized the same non-medication features as Model A but utilized medication features based on binary indicators. Two models were developed—one with no medication-based features (Model C) and one with only medication history features (Model D)—to ascertain the role that medications play in the model. Although medication did increase the positive predictability of MV, Fig 4 and Table 2 show that medication alone did not outperform medication with assessment features.

**Table 2:** Performance metrics of models.

Performance metrics using XGBoost	Model-A: Non-medication features + medication history features	Model-B: Non-medication features + medication indicator features	Model-C: Non-medication features only	Model-D: Medication history features only
AUROC	0.89	0.86	0.85	0.84
AUPRC	0.55	0.46	0.45	0.43
Sensitivity (recall)	0.47	0.43	0.47	0.56
Specificity	0.96	0.95	0.94	0.89
Positive predictive value (precision)	0.54	0.45	0.42	0.33
Negative predictive value	0.95	0.95	0.95	0.96
F1 score	0.50	0.44	0.44	0.41
Early warning time* (hours)	9.9 [4.2–69.2]	40.2 [11.0–134.3]	55.2 [14.1–151.1]	6.2 [3.6–38.3]

*Expressed as median [IQR]

AUROC: Area under receiver operating characteristic curve

AUPRC: area under precision-recall curve

**Fig 5:**
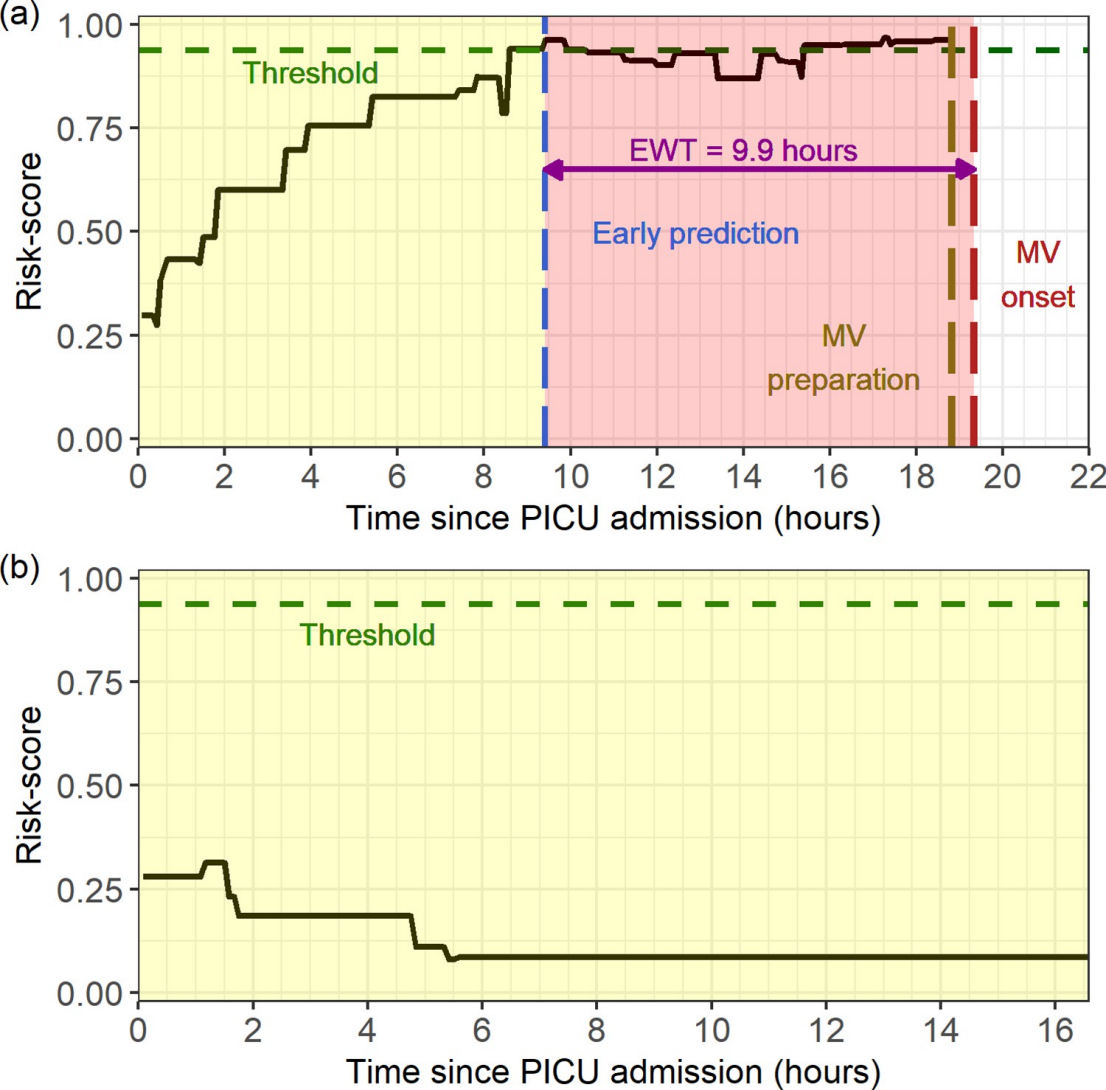
Time evolving risk-score. Time evolving risk-score of (a) a patient who received mechanical ventilation (b) a patient who did not receive mechanical ventilation.

**Fig 6:**
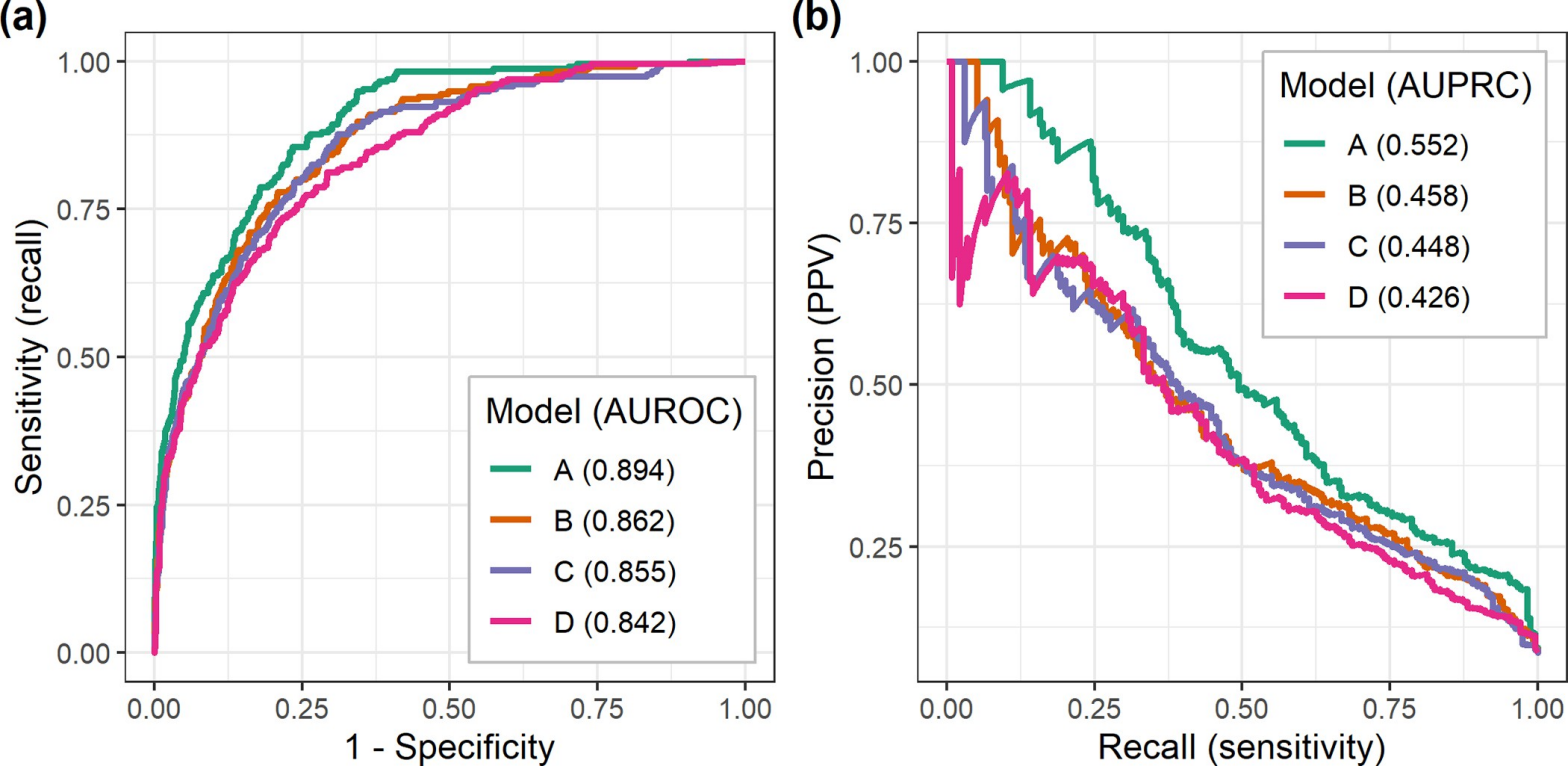
(a) Receiver operating characteristic (ROC) curves, and precision-recall (PR) curves for model A and model B. Model A consisted of non-medication features and CNN based medication history features. Model B consisted of non-medication features and medication indicator features.

Models A and B were trained using both XGBoost [25, 26] and Lasso-GLM [27, 43]. Of the two, XGBoost had higher area under precision-recall curve (AUPRC) (A: 0.55 vs 0.52, B: 0.46 vs 0.4) and positive predictive value (PPV) (A: 0.54 vs 0.51, B: 0.45 vs. 0.4) (S1 Table 3 in the S1 Appendix). Examples of risk scores produced over time by Model A for MV and no-MV cases are shown in Fig 5. The ROC and precision-recall (PR) curves for the two models are shown in Fig 6. Models A and B achieved median [IQR] area under ROC (AUROC) 0.89[0.89–0.9] vs. 0.86[0.85–0.87], AUPRC 0.52[0.51–0.56] vs 0.45[0.42–0.46] and PPV 0.51[0.48–0.53] vs. 0.4[0.35–0.46] during 10-fold cross-validation on the training set (see complete cross-validation performance metrics in Fig 4). Unless otherwise specified, the study’s final performance metrics apply to the held-out test set. All performance indicators for Models A, B, C, and D on the test set are shown in Table 2. Model A had the highest AUROC (0.89), AUPRC (0.55), and PPV (0.54). The EWT was 9.9[4.2–69.2] and 40.2[11.0–134.3] hours, respectively, for models A and B.

Unless otherwise specified, all results in the subsequent subsections are based on Model A, the model with the best overall performance.

### Risk-based patient grouping

**Fig 7:**
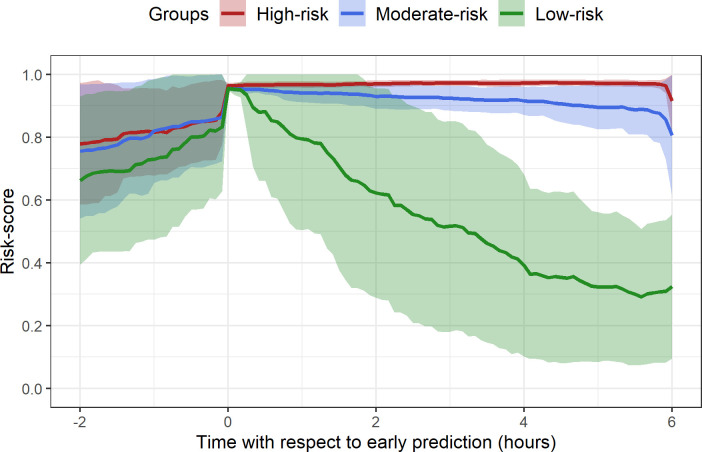
Risk groups obtained using K-NN classifier on test set using spectral clustering labels. Risk groups obtained using K-NN classifier on test set using spectral clustering labels obtained on training data as reference. The risk scores in this figure were generated from Model A. Solid lines represent the mean, and the shaded regions represent mean ± standard deviation across each group.

Natural groupings in the time-varying risk-score trajectories were observed after a patient crossed the EPP (Fig 7). Within 0–6 hours after passing the EPP in Model A, three distinct groups appeared using spectral clustering on the risk-score trajectories. These clusters were named as the low, medium, and high-risk groups based on their average risk-scores at EPP. The time-varying risk-scores across all positive predicted cases were less distinguishable 2–3 hours before EPP but showed a steady increase leading up to the time of prediction, and finally diverged into three distinct risk groups (Fig 7). Although the high and medium risk groups had consistently high risk-scores over time, the low-risk group had a significant decline in risk-score within 6 hours after EPP. The PPV increased and median EWT decreased across the low, medium, and high-risk groups (Table 3). Histograms of EWT for each risk group are shown in S1 Fig 1 in the S1 Appendix. The high-risk group with the highest risk-scores had a substantially higher PPV (0.92) as compared to other groups (0.55, 0.15) and the complete test set (0.53).

**Table 3:** Positive predictive values and early warning times across risk groups in test set.

Groups	Number of positive predictions	Positive Predictive Value (precision)	Early Warning Time* (hours)
High risk group	49	0.92	6 [3.2–39.5]
Moderate risk group	106	0.57	21.3 [5.1–111.8]
Low risk group	52	0.12	43.8 [14.3–66.1]

*Expressed as median [IQR] across all true positives

### Performance stratified by diagnostic categories

The prevalence of at least one recorded ICD diagnostic code was 55% across the included PICU encounters (99.7% in MV, 50.8% in no-MV). Baseline statistics on diagnostic categories are presented in S1 Table 4 in the S1 Appendix. The rates on incidence of diagnostic categories across risk groups are presented in S1 Table 5 in the S1 Appendix.

### Feature importance

XGBoost’s model-based global feature importance metric “Gain” and model-agnostic average absolute SHAP (Shapley Additive Explanation) values evaluated on training data are presented in Table 4. S1 Fig 1 in the S1 Appendix shows examples of local feature attributions using SHAP values before and after EPP in an MV case. The five features with highest observed average SHAP values across positive predicted cases evaluated at EPP are, in rank order: IV rate, age, PO rate, respiratory rate, and heart murmurs (S1 Fig 2 in S1 Appendix).

**Table 4:** Global feature importance for the 20 highest ranked features across the training set computed using XGBoost gain metric and average absolute SHAP value.

Feature rank	Feature name	Gain (XGBoost)	Feature name	Average |SHAP| value
Model-A (non-medication features + medication history features from pre-trained CNN)				
1	IV rate	0.138	IV rate	0.438
2	Age	0.072	Age	0.293
3	medhist2	0.054	PO rate	0.250
4	medhist9	0.043	medhist2	0.194
5	medhist7	0.041	medhist12	0.149
6	HeartSounds.Murmur	0.037	HeartSounds.Murmur	0.145
7	PO rate	0.036	RightResponse.Brisk	0.144
8	medhist3	0.032	Bilirubin	0.140
9	Bilirubin	0.030	Height	0.124
10	Height	0.029	Resp	0.121
11	Resp	0.021	medhist7	0.116
12	Weight	0.018	medhist9	0.115
13	Carbon dioxide (blood)	0.018	medhist3	0.104
14	FiO2	0.018	Alkaline Phosphatase	0.104
15	Alkaline Phosphatase	0.017	FiO2	0.103
16	RightResponse.NotMeasured	0.016	RightResponse.NotMeasured	0.095
17	RDW	0.016	DBP	0.090
18	RespiratoryEffortORPattern.Tachypneic	0.016	Chloride	0.087
19	RightResponse.Brisk	0.015	PCO2 Venous	0.087
20	medhist12	0.015	RDW	0.079
Model-B (non-medication features + medication indicator features)				
1	IV rate	0.192	IV rate	0.548
2	Age	0.082	PO rate	0.349
3	PO rate	0.064	Age	0.296
4	HeartSounds.Murmur	0.047	RightResponse.Brisk	0.178
5	Bilirubin	0.038	Bilirubin	0.157
6	Height	0.034	HeartSounds.Murmur	0.143
7	Resp	0.025	Height	0.140
8	RightResponse.Brisk	0.024	Resp	0.122
9	Alkaline Phosphatase	0.023	Chloride	0.112
10	RespiratoryEffortORPattern.Tachypneic	0.021	Alkaline Phosphatase	0.107
11	GCS Eye	0.021	RightResponse.NotMeasured	0.084
12	Weight	0.020	RDW	0.082
13	Chloride	0.020	DBP	0.081
14	Carbon dioxide (blood)	0.018	Calcium	0.080
15	Creatinine	0.018	PCO2 Venous	0.080
16	RDW	0.018	GCS Eye	0.079
17	RightResponse.NotMeasured	0.016	BUN by Creatinine ratio	0.072
18	FiO2	0.016	Creatinine	0.071
19	C-Reactive Protein	0.015	RespiratoryEffortORPattern.Tachypneic	0.071
20	RespiratoryEffortORPattern.Labored	0.015	State.Alert	0.069

### Model B variations

A longer EWT is often preferred by clinicians to have enough time to potentially alter a patient’s clinical course. Since Model B had a considerably longer median EWT than Model A, a subsequent analysis on variations of Model B was conducted to explore if it could improve the overall positive predictive value, AUROC, and AUPRC. Two different prediction schemes were tested: Model B1 with a dual threshold and Model B2 using a waiting period around the EPP. The detailed performance of Models B1 and B2 are presented in the S1 Table 6 in S1 Appendix. Model B1 showed a marginal improvement over Model B in AUROC (0.88 vs 0.86) and AUPRC (0.49 vs 0.46), but slightly lesser PPV (0.42 vs 0.45). The median EWT with the dual-threshold method with 6-hour period between two thresholds (Model B1) was the highest among all the variants of Model B (Model B: 40.25 hours, Model B1: 60.1 hours, and Model B2: 59.7 hours).

### Prediction with PEWS

Pediatric Early Warning Score (PEWS) [44] is a commonly used scoring system in pediatric inpatient medical units to identify patients at risk for patient deterioration. This nursing and respiratory therapist driven scoring system is based on a patients’ behavior (alert, irritable, baseline etc.), cardiovascular and respiratory findings such as heart rate, respiratory rate and need for supplemental oxygen [45]. While PEWS was developed for use on the medical floor, investigators have used this scoring system on intermediate medical units. Since there are no published early warning prediction models, the PEWS scoring system was evaluated on the dataset. The median [IQR] of maximum Pediatric Early Warning Score (PEWS) was 4 [3–5] for MV cases evaluated until MV onset and 3 [2–4] during entire LOS among no-MV cases. Maximum PEWS until MV onset achieved 0.6 AUROC, 0.13 AUPRC, and by using the optimal threshold from training set, PEWS > 5 classifier yielded 0.39 sensitivity, 0.77 specificity, 0.13 PPV, 0.93 negative predictive value (NPV), 0.2 F1 score, and median [IQR] EWT of 33.7 [8.8–84.9] hours. Daily-PEWS (maximum PEWS over 24-hour intervals) predicted MV within the following 24 hours with 0.67 AUROC, 0.02 AUPRC, and the classifier daily-PEWS > 6 yielded 0.15 sensitivity, 0.94 specificity, 0.03 PPV, 0.99 NPV, 0.05 F1 score and median [IQR] lead time between MV onset and the first instance of PEWS>6 on the day prior to MV onset was 25 [21–31.6] hours.

## Discussion

### Predictive performance of models

Predicting which patients are likely to need MV can be done using standard assessments that are frequently recorded in PICU patients along with medication history. Based on several test set performance metrics, Model A was found to be the best of the six proposed models. The models show that for specific medications, convolutional neural networks are more predictive than traditional binary indicators. Model C’s (which lacked any medication-based features) PPV was the lowest of the models A through D, indicating that the medication data is a significant predictor on its own. Additionally, even without any additional non-medication features, Model D, which only uses medication history features, performed reasonably well. Based on the combination or administration order of the given medications, a clinician can undoubtedly infer the clinical state of a patient. For instance, a patient receiving a higher or more frequent dose of a diuretic could infer the patient’s fluid status without any other clinical features. Since the drug can also be used to treat high blood pressure, heart failure, improve urine output, etc., its presence alone is less beneficial. In contrast to using individual binary indicators, using the natural language of medication history (increased, decreased, combination with other medications) better represents a patient’s clinical condition. The study found that Model A, which incorporates clinical assessments and the natural language of medication histories, performed the best. Regardless of EMR, Model D, which uses universal medication usage language, may be adopted in the future by institutions and may provide enough positive predictive value to assist clinicians in monitoring patients at risk for mechanical ventilation.

On Model A, XGBoost outperformed lasso-GLM in terms of AUPRC, PPV, and median EWT. XGBoost, as a non-linear technique, also has the advantage of being able to discover more complex non-linear relationships between predictors and outcomes, and unlike most other methods, including lasso-GLM, it does not require explicit imputation of missing values. To build each decision tree, XGBoost first uses information from the observed data. It then assigns a default direction for missingness in each predictor at each split in order to minimize training error [26]. XGBoost takes missingness into account at each decision branch, rather than as a constant mean or median, resulting in a better fit in "messy" datasets.

### Potential benefits of risk grouping

When the early prediction point (EPP) is reached, groups of patient subtypes emerged. The spectral clustering of risk-score trajectories within a 6-hour period after EPP revealed low, medium, and high-risk groups with an increasing trend in PPV. The mean risk-score trajectory in the high and medium risk groups showed considerably higher risk-scores after EPP, which could be indicative of sustained aberration from normal physiologic state among the more severe cases. The low-risk group, on the other hand, had the highest number of false positives and a mean trajectory that gradually declined over time. The high-risk group had >0.92 PPV, providing further confidence to the positive predictions. Bose et al. [39] and Liu et al. [40] found similar distinct clusters of risk-score trajectories with corresponding different positive predictive values/risk group for the prediction of multiple organ dysfunction and septic shock in PICU patients. The histogram of early warning time (EWT) across risk groups revealed an inverse relationship between median EWT and group severity. Since clustering was applied to 6 hours of continuously observed risk scores, a median EWT of 6 hours in the high-risk group might only allow time to prepare for intubation. The proposed scheme not only allows continued monitoring of patients who have been predicted to likely require MV, but also assigns varying confidence levels on predictions based on PPV associated with that risk group. Therefore, the risk grouping approach could significantly aid hospital caregivers in triaging patients based on severity of illness and in efficient utilization of hospital resources for patient management.

### Association with diagnostic categories

Respiratory diseases, multiple organ dysfunction, sepsis, prematurity, immune system disorders, and hematological/oncological disorders are the most common comorbidities associated with ARF and ARDS in the pediatric population [46–51]. The study observed corroborating evidence from statistically significant associations between MV and some of these diseases. In high-risk groups, the following International Classification of Diseases (ICD-10) groups were more prevalent: health services, cardiovascular, respiratory, congenital diseases, congenital heart diseases, problems originating in the perinatal period, and respiratory failure. The presence of co-morbidities was linked to an increased risk of mechanical ventilation. Certain conditions, such as congenital diseases, prematurity history, and even the presence of home equipment (ICD-10- health services), were more common in the high-risk group. Chronic conditions alone did not increase risk since hematologic condition or neoplasm were more common in the low-risk group.

### Global and local feature importance

Strikingly, 5 of the of the top 20 globally important features were medication history features. This strengthens the rationale for using a language-processing model to depict medication history to improve the model’s predictive performance. The high concordance between the top 20 features from both methods indicates that the importance of global features is consistent across the two methods. In addition, SHAP values can also explain local feature attributions for individual predictions on test data.

### Comparison with PEWS

PEWS is the most widely used scoring system for continuous assessment of subject’s inpatient clinical status. A 2017 retrospective study by Dean et al. presented a classifier to predict the occurrence of critical PICU interventions (intubation, high flow nasal cannula, NIV, inotropes, and aggressive fluid administration) within 12 hours of PICU transfer based on 24-hour maximum modified PEWS with an AUROC of 0.88 and PEWS ≥ 5 yielded 38% sensitivity and 99% specificity in the general medicine subgroup of patients [52]. Lockwood et al. evaluated AutoPEWS (an automated adaption of PEWS from EHR) predicted critical deterioration events within the prior 24 hours with 0.78 AUROC [53]. However, in the dataset, both maximum PEWS and daily-PEWS based classifiers had lower AUROC (0.6, 0.65) and very low PPV (0.13, 0.02). This disparity in performance across PEWS-based classifiers could be attributed to differences in patient characteristics across studies, changes to PEWS evaluation criteria, and the fact that prior studies frequently evaluated PEWS only prior to PICU admission, whereas this study evaluated PEWS on patients who had already been admitted to PICU. Dean et al. also reported the median[IQR] lead time between observed maximum PEWS and a critical event was 2.0 [1.0–4.7] hours [52], which is substantially less than the EWT of Model A (9.9 [4.2–69.2] hours) and also smaller than the EWT for the identified high risk group (6 [3.2–39.5] hours). This study found neither maximum PEWS nor daily-PEWS to be a strong independent predictor of MV within the dataset.

### Limitations

This study used clinical assessments and manually entered data fields in addition to automatically imported discrete values such as vital signs. The use of human entered values can lead to transcription errors and errors in accuracy of timing of events. Forty-five percent of all patients had no available ICD-10 diagnostic codes in the retrospective dataset due to a change in billing software. Thus, the association of various diagnostic categories with MV vs no-MV outcome and across the stratified risk groups should be validated in future studies. Lastly, due to the single-center retrospective nature of the study dataset, future multicenter prospective studies would increase the applicability of these models on general PICU patient population across diverse health systems.

### Research contributions and conclusions

Most of the existing work on pediatric acute respiratory failure is focused on the prediction of prolonged mechanical ventilation [13, 54–58] and risk factors associated with failure of non-invasive ventilation strategies [3, 7–12]. However, no existing studies have developed a prediction model for a general PICU population encompassing a wide range of comorbidities and for all patients, irrespective of prior non-invasive ventilation status. Hence, this study was aimed at creating a widely applicable model for making early predictions of the need for mechanical ventilation in critically ill pediatric patients. The distinct advantages of the study in comparison to prior studies are: (a) the continuous risk-score evaluation allows for continuous monitoring of patient state; (b) EPP enables early alert for healthcare providers to prescribe an appropriate treatment regimen based on the patient’s physiological state at that time; and (c) the proposed risk stratification method would allow for identification of different risk groups after observed EPP, which can help identify a high-risk subgroup that has an extremely high likelihood of intubation.

Some of the common risk factors associated with failure of non-invasive ventilation in children reported by prior studies [59–65] include higher FiO2, SpO2/FiO2 ratio, respiratory rate oxygenation index (ratio of SpO2/FiO2 to respiratory rate), heart rate, respiratory rate, younger age, and blood pH. The global variable importance of Model A also suggests corroborating evidence that SpO2, FiO2, age, respiratory rate, tachypnea, etc. were among the top 20 predictors of MV. The top predictors in Model A also had a good agreement with the important predictors of ARF in adult COVID-19 patients, as reported by Ferrari et al. in their gradient boosting machine (GBM)-based model [66]. Not many studies have reported medication-based strong predictors of MV; one such study by Suessman et al. [63] showed that use of bronchodilators was associated with a reduced incidence of intubation. The study has successfully demonstrated that medical history features were important predictors of MV in the dataset, and the proposed method of using medication history instead of indicator variables for individual medications is an extremely valuable method to capture the physiologic state of a patient.

In conclusion, this study demonstrates that clinical assessments and medication history can be effectively used to drive a machine learning-based prediction model aimed at detecting the need for MV early, with a median EWT of 9.9 hours. Furthermore, the risk grouping methodology can help with continuous patient monitoring and stratification of patients into subgroups to allow risk-based intervention.

## Supporting information

S1 Appendix (DOCX)
