# Supplementary material for: A data-driven model for early prediction of need for invasive mechanical ventilation in pediatric intensive care unit patients
Source: PLoS One. 2023 Aug 4;18(8):e0289763. doi: 10.1371/journal.pone.0289763 (PMC10403092; doi:10.1371/journal.pone.0289763)
Supplement: S1 Appendix — (DOCX) [file pone.0289763.s001.docx]

**A data-driven model for early prediction of need for invasive mechanical ventilation in pediatric intensive care unit patients.**

**Authors**

Sanjukta N. Bose, Andrew Defante, Joseph L. Greenstein, Gabriel G. Haddad, Julie Ryu, and Raimond L. Winslow

**Online Supplement**


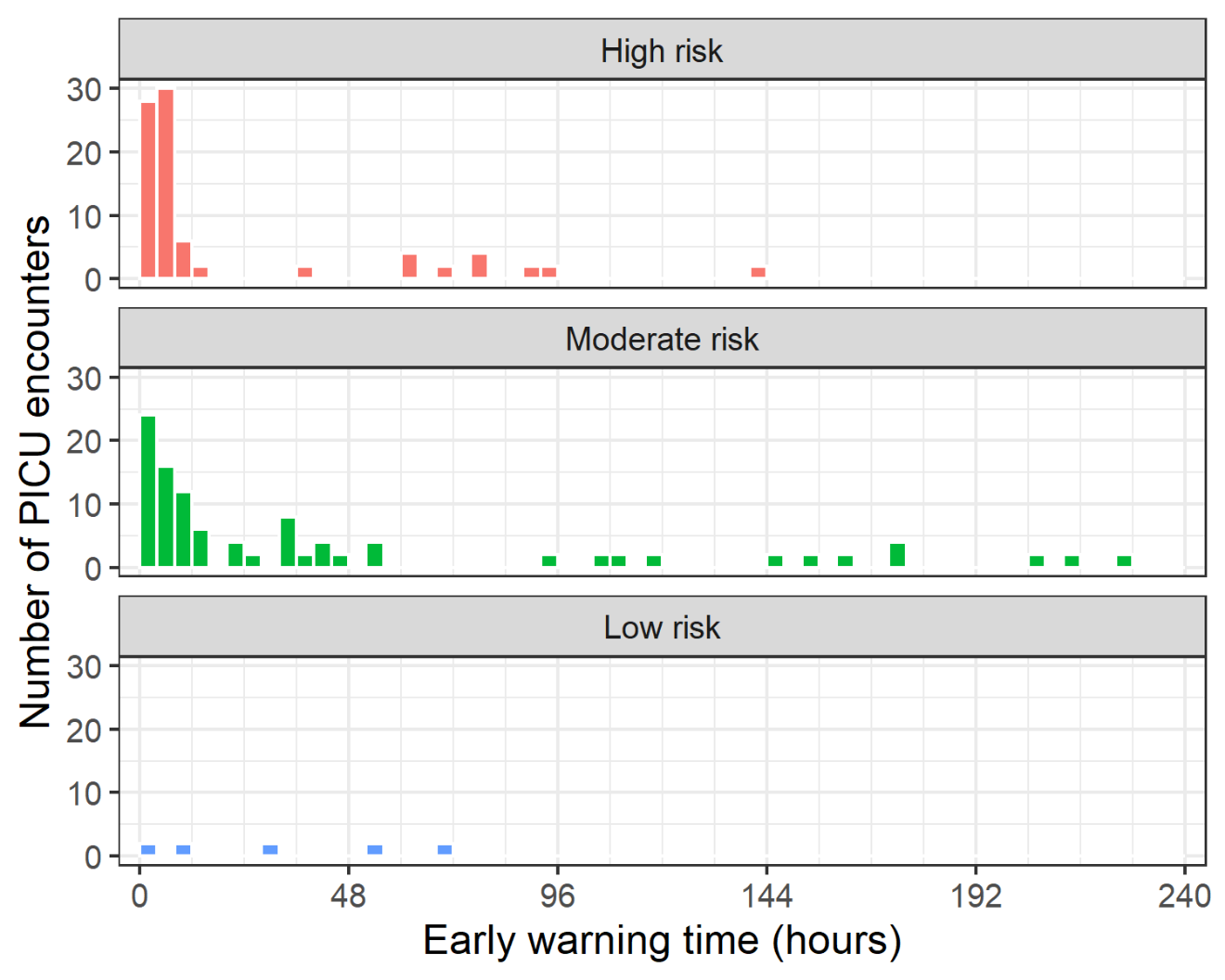


**S1 Fig 1.** **Histogram of early warning times (EWTs) for different risk-groups within test set obtained from Model A**. The above histogram excludes 20 PICU encounters with longer than 240 hours EWT for the ease of visualization.


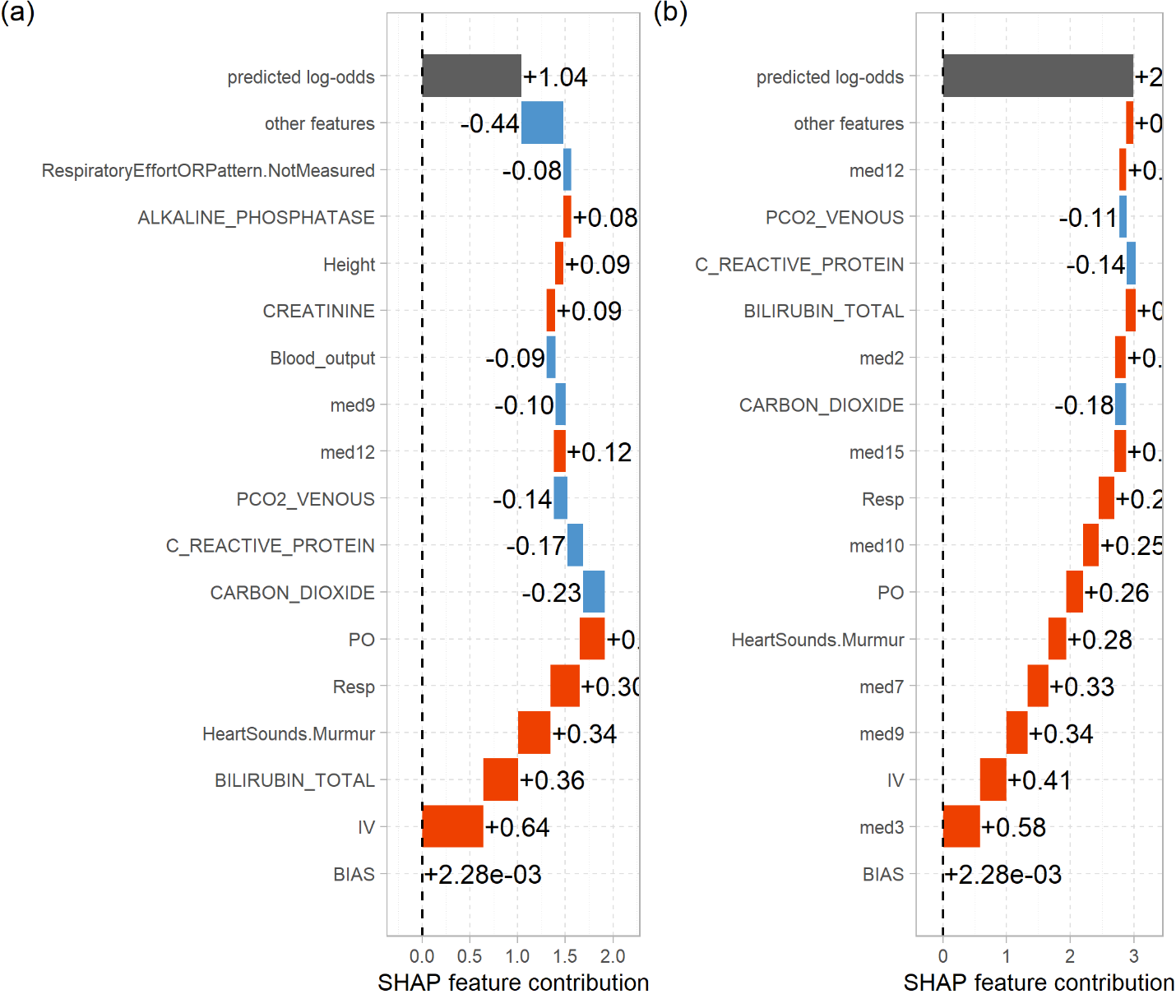


**S1 Fig 2.** **Local feature attributions using SHAP values** in (a) 5 before early prediction and (b) at the time of early prediction in an MV case.

**S1 Table 1. List of medications used as indicator based medication features**

| Feature name | Medications |
| --- | --- |
| Med01 | MIDAZOLAM HCL 2 MG/2ML IJ SOLN |
| Med02_c^*^ | DOPAMINE IV INFUSION |
| Med03_c^*^ | MILRINONE IV INFUSION |
| Med04 | FUROSEMIDE 10 MG/ML IJ SOLN |
| Med05 | MEROPENEM 20 MG/ML IVPB (COMPOUNDED) |
| Med06 | LIDOCAINE 4 % EX CREA |
| Med07 | ACETAMINOPHEN 160 MG/5ML PO SUSP |
| Med08 | CHOLECALCIFEROL 400 UNIT/ML PO LIQD |
| Med09 | ZINC OXIDE 40 % EX OINT |
| Med10 | METHYLPREDNISOLONE SODIUM SUCC 125 MG IJ SOLR (62.5 MG/ML) |
| Med11 | THROMBIN TOPICAL (RECOMBINANT) 5000 UNITS EX SOLR |
| Med12_c^*^ | EPINEPHRINE IV INFUSION |
| Med13 | LORAZEPAM 2 MG/ML IJ SOLN |
| Med14_c^*^ | DEXMEDETOMIDINE 2 & 4 MCG/ML IV INFUSION |
| Med15_c^*^ | NITROPRUSSIDE IV INFUSION |
| Med16 | SODIUM BICARBONATE 8.4 % IV SOLN |
| Med17 | FENTANYL CITRATE 0.05 MG/ML IJ SOLN |
| Med18 | VECURONIUM BROMIDE 10 MG IV SOLR |
| Med19 | NICU FLUSH 1/2 NS + HEPARIN 1 UNIT/ML VIAL |
| Med20_c^*^ | CALCIUM CHLORIDE 8 20 50 MG/ML IV INFUSION |
| Med21 | IOVERSOL 68 % IV SOLN |
| Med22 | FUROSEMIDE 10 MG/ML PO SOLN |
| Med23 | VANCOMYCIN DILUTE 5 MG/ML IVPB IN D5W |
| Med24 | CRITIC-AID THICK MOIST BARRIER EX PSTE |
| Med25 | ROCURONIUM BROMIDE 50 MG/5ML IV SOLN |
| Med26 | CEFAZOLIN 1000 MG IN 1000 ML 0.9 % SODIUM CLORIDE (OPTIME) |
| Med27 | LABELS FOR BREASTMILK |

^*^_c suffix indicates continuously intravenously administered medications

**S1 Table 2. List of all non-medication features.**

| **Feature** | **Description** | **Type** | **Units** |
| --- | --- | --- | --- |
| GCS Eye | Component of Glasgow Coma Score (GCS) | Ordinal | - |
| GCS Motor | Component of GCS | Ordinal | - |
| GCS Total | Total GCS | Ordinal | - |
| GCS Verbal | Component of GCS | Ordinal | - |
| Degree of HOB | Degree of head of bed | Numeric | degrees |
| IV rate | Rate of intravenous fluid input normalized by patient weight | Numeric (rate calculated) | mL/kg/min |
| PO rate | Rate of orally provided input normalized by patient weight | Numeric (rate calculated) | mL/kg/min |
| Blood input rate | Rate of blood input normalized by patient weight | Numeric (rate calculated) | mL/kg/min |
| Urine output rate | Rate of urine output normalized by patient weight | Numeric (rate calculated) | mL/kg/min |
| Blood output rate | Rate of blood output normalized by patient weight | Numeric (rate calculated) | mL/kg/min |
| PO2 Arterial | Arterial partial pressure of O2 | Numeric | mm Hg |
| PO2 Venous | Venous partial pressure of O2 | Numeric | mm Hg |
| PCO2 Arterial | Arterial partial pressure of O2 | Numeric | mm Hg |
| PCO2 Venous | Venous partial pressure of O2 | Numeric | mm Hg |
| pH Arterial | Arterial blood pH | Numeric | - |
| pH Venous | Venous blood pH | Numeric | - |
| Albumin | Albumin | Numeric | g/dL |
| Albumin by Globulin ratio | Albumin by Globulin ratio | Numeric | - |
| Alkaline Phosphatase | Alkaline Phosphatase | Numeric | U/L |
| Alanine Aminotransferase | Alanine Aminotransferase | Numeric | U/L |
| Anion gap | Anion gap | Numeric | mmol/L |
| Aspartate Aminotransferase | Aspartate Aminotransferase | Numeric | U/L |
| Bilirubin | Bilirubin | Numeric | mg/dL |
| BUN by Creatinine ratio | Blood Urea Nitrogen by Creatinine ratio | Numeric | - |
| C-Reactive Protein | C-Reactive Protein | Numeric | mg/dL |
| Calcium | Calcium | Numeric | mg/dL |
| Carbon dioxide (blood) | Carbon dioxide (blood) | Numeric | mmol/L |
| Chloride | Chloride | Numeric | mmol/L |
| Creatinine | Creatinine | Numeric | mg/dL |
| Glucose | Glucose | Numeric | mg/dL |
| Hematocrit | Hematocrit | Numeric | % |
| Hemoglobin | Hemoglobin | Numeric | g/dL |
| MCH | Mean Corpuscular Hemoglobin | Numeric | pg |
| MCHC | Mean Corpuscular Hemoglobin Concentration | Numeric | % |
| MCV | Mean Corpuscular Volume | Numeric | fL |
| Platelet count | Platelet count | Numeric | fL |
| Potassium | Potassium | Numeric | mmol/L |
| RDW | Red blood cell distribution width | Numeric | RDW Unit |
| RBC count | Red blood cell count | Numeric | - |
| Sodium | Sodium | Numeric | mmol/L |
| BUN | Blood Urea Nitrogen | Numeric | mg/dL |
| WBC count | White blood cell count | Numeric | - |
| FiO2 | Fraction of inspired oxygen | Numeric | % |
| O2 flow rate | Oxygen flow rate | Numeric | L/min |
| INO ppm | Inhaled nitric oxide | Numeric | ppm |
| Speciality gas | Specialty gas given through ventilation (1=given, 0=not given) | Binary | - |
| Height | Height | Numeric | inches |
| Pulse | Heart rate/pulse | Numeric | /min |
| Resp | Respiratory rate | Numeric | /min |
| SpO2 | Peripheral oxygen saturation (pulse oximetry) | Numeric | % |
| Temperature | Body temperature | Numeric | F |
| Weight | Weight | Numeric | lbs |
| SBP | Systolic Blood Pressure | Numeric | mm Hg |
| DBP | Diastolic Blood Pressure | Numeric | mm Hg |
| Gender | Gender (1=Male, 0=Female) | Binary | - |
| Age | Age | Numeric | years |
| Specialty_Gas.NotMeasured  Specialty_Gas.Heliox  Specialty_Gas.INO  Specialty_Gas.Nitrogen | Specialty gas given through ventilation | Categorical | - |
| BiPAP_Mode.NotMeasured  BiPAP_Mode.Spontaneous_Timed  BiPAP_Mode.CPAP | BiPAP mode for non-invasive ventilation | Categorical | - |
| stat.RA  stat.O2  stat.MV  stat.NIV | Respiratory support status (RA: room air, O2: supplemental oxygen, NIV: non-invasive ventilation, MV: invasive mechanical ventilation) | Categorical | - |
| State.NotMeasured  State.Responds_to_stimulation  State.Lethargic  State.Arousable  State.Follows_commands  State.Drowsy  State.Sleeping  State.Awake  State.Alert  State.Sedated_note_if_arousable  State.Unarousable  State.Crying  State.Restless  State.Difficult_to_arouse  State.Neuromuscular_blockade  State.Other  State.Jittery  State.Irritable  State.Confused | Behavioral state of patient | Categorical | - |
| LeftResponse.NotMeasured  LeftResponse.Brisk  LeftResponse.Sluggish  LeftResponse.Hippus  LeftResponse.Non_reactive  LeftResponse.Swollen_shut | Left pupil response | Categorical | - |
| RightResponse.NotMeasured  RightResponse.Brisk  RightResponse.Sluggish  RightResponse.Hippus  RightResponse.Non_reactive  RightResponse.Swollen_shut | Right pupil response | Categorical | - |
| ChestExpansionORAssessment.NotMeasured  ChestExpansionORAssessment.Adequate  ChestExpansionORAssessment.Equal_bilaterally  ChestExpansionORAssessment.Trachea_midline  ChestExpansionORAssessment.Shallow  ChestExpansionORAssessment.Right_Left  ChestExpansionORAssessment.Left_Right  ChestExpansionORAssessment.Trachea_deviates_left  ChestExpansionORAssessment.Trachea_deviates_right  ChestExpansionORAssessment.HFOV_Chest_vibration_equal  ChestExpansionORAssessment.HFOV_Wiggle | Chest expansion or assessment | Categorical | - |
| Cough.NotMeasured  Cough.Absent  Cough.Dry  Cough.Nonproductive  Cough.Weak  Cough.Intermittent  Cough.Moist  Cough.Strong  Cough.Productive_Effective  Cough.Frequent  Cough.Ineffective  Cough.Intractable  Cough.Paroxysmal | Cough | Categorical | - |
| RespiratoryEffortORPattern.NotMeasured  RespiratoryEffortORPattern.Tachypneic  RespiratoryEffortORPattern.Kussmal  RespiratoryEffortORPattern.Adequate  RespiratoryEffortORPattern.Labored  RespiratoryEffortORPattern.Intermittent  RespiratoryEffortORPattern.Dyspnea_with_exertion  RespiratoryEffortORPattern.Regular  RespiratoryEffortORPattern.Unlabored  RespiratoryEffortORPattern.Shallow  RespiratoryEffortORPattern.Even  RespiratoryEffortORPattern.Ventilated  RespiratoryEffortORPattern.Accessory_muscle_use  RespiratoryEffortORPattern.Periodic_breathing  RespiratoryEffortORPattern.Sedated  RespiratoryEffortORPattern.Medically_paralyzed  RespiratoryEffortORPattern.Nasal_congestion  RespiratoryEffortORPattern.Weak  RespiratoryEffortORPattern.Irregular  RespiratoryEffortORPattern.Absent  RespiratoryEffortORPattern.Dyspena_at_rest  RespiratoryEffortORPattern.Apneic  RespiratoryEffortORPattern.Agonal  RespiratoryEffortORPattern.Orthopneic  RespiratoryEffortORPattern.Cheyne_Stokes | Respiratory effort or pattern | Categorical | - |

**S1 Table 3. Comparison of test set performance of models A and B trained using XGBoost and lasso-GLM.**

| Performance metrics | Model-A:  Non-medication features + medication history features | | Model-B:  Non-medication features + medication indicator features | |
| --- | --- | --- | --- | --- |
|  | **XGBoost** | **Lasso-GLM** | **XGBoost** | **Lasso-GLM** |
| AUROC | 0.894 | 0.886 | 0.862 | 0.842 |
| AUPRC | 0.55 | 0.52 | 0.46 | 0.40 |
| Sensitivity (recall) | 0.47 | 0.47 | 0.43 | 0.49 |
| Specificity | 0.96 | 0.96 | 0.95 | 0.93 |
| Positive predictive value (precision) | 0.54 | 0.51 | 0.45 | 0.40 |
| Negative predictive value | 0.95 | 0.95 | 0.95 | 0.95 |
| F1 score | 0.50 | 0.49 | 0.44 | 0.44 |
| Early warning time^*^  (hours) | 9.9 [4.2 – 69.2] | 5.8 [2.4 – 41.5] | 40.2 [11.0 – 134.3] | 24.0 [4.1 – 88.2] |

^*^Expressed as median [IQR]
AUROC: Area under receiver operating characteristic curve
AUPRC: area under precision-recall curve

**S1 Table 4. Prevalence of diagnostic categories in the study population.** For each diagnostic category, up to 5 most prevalent diagnostic codes with at least 1% overall prevalence or a difference of 5% points between MV and no-MV groups are presented, and in cases where all codes had less than 1% prevalence, only the highest prevalence code within each category is presented.

| **Diagnostic category** | **ICD-10**  **Diagnostic code(s)** | **Description** | **N (%) in dataset**  (Total = 13651) | **N (%) in each group** | | **p-value^*^** |
| --- | --- | --- | --- | --- | --- | --- |
|  |  |  |  | **No-MV**  (Total = 12475) | **MV**  (Total = 1176) |  |
| **Abnormal clinical findings** | **R00 - R99** |  | **4850 (35.5%)** | **3954 (31.7%)** | **896 (76.2%)** | **<0.001** |
|  | R06.03 | Acute respiratory distress | 1598 (11.7%) | 1393 (11.2%) | 205 (17.4%) | <0.001 |
|  | R09.02 | Hypoxemia | 1209 (8.9%) | 1021 (8.2%) | 188 (16%) | <0.001 |
|  | R50.9 | Fever, unspecified | 941 (6.9%) | 772 (6.2%) | 169 (14.4%) | <0.001 |
|  | R63.3 | Feeding difficulties | 362 (2.7%) | 207 (1.7%) | 155 (13.2%) | <0.001 |
|  | R06.82 | Tachypnea, not elsewhere classified | 359 (2.6%) | 312 (2.5%) | 47 (4%) | 0.004 |
| **Respiratory** | **J00 - J99, Q30 - Q34, P22 - P28, R04 - R07, R09** |  | **4135 (30.3%)** | **3416 (27.4%)** | **719 (61.1%)** | **<0.001** |
|  | R06.03 | Acute respiratory distress | 1598 (11.7%) | 1393 (11.2%) | 205 (17.4%) | <0.001 |
|  | R09.02 | Hypoxemia | 1209 (8.9%) | 1021 (8.2%) | 188 (16%) | <0.001 |
|  | J18.9 | Pneumonia, unspecified organism | 603 (4.4%) | 477 (3.8%) | 126 (10.7%) | <0.001 |
|  | J98.4 | Other disorders of lung | 436 (3.2%) | 329 (2.6%) | 107 (9.1%) | <0.001 |
|  | R06.82 | Tachypnea, not elsewhere classified | 359 (2.6%) | 312 (2.5%) | 47 (4%) | 0.004 |
| **Cardiovascular** | **I00 - I99, Q20 - Q28, R00, R01, R03** |  | **3272 (24%)** | **2473 (19.8%)** | **799 (67.9%)** | **<0.001** |
|  | R00.0 | Tachycardia, unspecified | 322 (2.4%) | 260 (2.1%) | 62 (5.3%) | <0.001 |
|  | Q21.1 | Atrial septal defect | 312 (2.3%) | 238 (1.9%) | 74 (6.3%) | <0.001 |
|  | Q21.0 | Ventricular septal defect | 284 (2.1%) | 149 (1.2%) | 135 (11.5%) | <0.001 |
|  | Q23.4 | Hypoplastic left heart syndrome | 190 (1.4%) | 140 (1.1%) | 50 (4.3%) | <0.001 |
|  | Q25.1 | Coarctation of aorta | 168 (1.2%) | 80 (0.6%) | 88 (7.5%) | <0.001 |
| **Health services (for non-disease encounters)** | **Z00 - Z99** |  | **2762 (20.2%)** | **1968 (15.8%)** | **794 (67.5%)** | **<0.001** |
|  | Z98.890 | Other specified postprocedural states | 613 (4.5%) | 380 (3%) | 233 (19.8%) | <0.001 |
|  | Z93.1 | Gastrostomy status | 597 (4.4%) | 411 (3.3%) | 186 (15.8%) | <0.001 |
|  | Z74.2 | Need for assistance at home and no other household member able to render care | 402 (2.9%) | 270 (2.2%) | 132 (11.2%) | <0.001 |
|  | Z87.74 | Personal history of (corrected) congenital malformations of heart and circulatory system | 377 (2.8%) | 202 (1.6%) | 175 (14.9%) | <0.001 |
|  | Z51.5 | Encounter for palliative care | 244 (1.8%) | 169 (1.4%) | 75 (6.4%) | <0.001 |
| **Congenital diseases** | **Q00 - Q99** |  | **2574 (18.9%)** | **1934 (15.5%)** | **640 (54.4%)** | **<0.001** |
|  | Q21.1 | Atrial septal defect | 312 (2.3%) | 238 (1.9%) | 74 (6.3%) | <0.001 |
|  | Q21.0 | Ventricular septal defect | 284 (2.1%) | 149 (1.2%) | 135 (11.5%) | <0.001 |
|  | Q90.9 | Down syndrome, unspecified | 228 (1.7%) | 165 (1.3%) | 63 (5.4%) | <0.001 |
|  | Q23.4 | Hypoplastic left heart syndrome | 190 (1.4%) | 140 (1.1%) | 50 (4.3%) | <0.001 |
|  | Q25.1 | Coarctation of aorta | 168 (1.2%) | 80 (0.6%) | 88 (7.5%) | <0.001 |
| **Congenital heart diseases** | **Q20 - Q28** |  | **1681 (12.3%)** | **1200 (9.6%)** | **481 (40.9%)** | **<0.001** |
|  | Q21.1 | Atrial septal defect | 312 (2.3%) | 238 (1.9%) | 74 (6.3%) | <0.001 |
|  | Q21.0 | Ventricular septal defect | 284 (2.1%) | 149 (1.2%) | 135 (11.5%) | <0.001 |
|  | Q23.4 | Hypoplastic left heart syndrome | 190 (1.4%) | 140 (1.1%) | 50 (4.3%) | <0.001 |
|  | Q25.1 | Coarctation of aorta | 168 (1.2%) | 80 (0.6%) | 88 (7.5%) | <0.001 |
|  | Q24.9 | Congenital malformation of heart, unspecified | 161 (1.2%) | 89 (0.7%) | 72 (6.1%) | <0.001 |
| **Metabolic disorders** | **E00 - E89** |  | **1507 (11%)** | **1168 (9.4%)** | **339 (28.8%)** | **<0.001** |
|  | E86.0 | Dehydration | 352 (2.6%) | 296 (2.4%) | 56 (4.8%) | <0.001 |
|  | E87.2 | Acidosis | 161 (1.2%) | 120 (1%) | 41 (3.5%) | <0.001 |
| **Neurologic disorders** | **G00 - G99** |  | **1441 (10.6%)** | **1163 (9.3%)** | **278 (23.6%)** | **<0.001** |
|  | G40.909 | Epilepsy, unspecified, not intractable, without status epilepticus | 202 (1.5%) | 167 (1.3%) | 35 (3%) | <0.001 |
|  | G91.9 | Hydrocephalus, unspecified | 168 (1.2%) | 137 (1.1%) | 31 (2.6%) | <0.001 |
| **Hematologic and immune disorders** | **D50 - D89** |  | **1332 (9.8%)** | **979 (7.8%)** | **353 (30%)** | **<0.001** |
|  | D64.9 | Anemia, unspecified | 299 (2.2%) | 218 (1.7%) | 81 (6.9%) | <0.001 |
|  | D69.6 | Thrombocytopenia, unspecified | 193 (1.4%) | 132 (1.1%) | 61 (5.2%) | <0.001 |
|  | D70.9 | Neutropenia, unspecified | 142 (1%) | 101 (0.8%) | 41 (3.5%) | <0.001 |
|  | D63.1 | Anemia in chronic kidney disease | 137 (1%) | 114 (0.9%) | 23 (2%) | 0.002 |
| **Injury/trauma** | **S00 - T88** |  | **1210 (8.9%)** | **1004 (8%)** | **206 (17.5%)** | **<0.001** |
|  | T45.1X5A | Adverse effect of antineoplastic and immunosuppressive drugs, initial encounter | 143 (1%) | 108 (0.9%) | 35 (3%) | <0.001 |
| **Gastro-intestinal** | **K00 - K95** |  | **1069 (7.8%)** | **765 (6.1%)** | **304 (25.9%)** | **<0.001** |
|  | K21.9 | Gastro-esophageal reflux disease without esophagitis | 259 (1.9%) | 164 (1.3%) | 95 (8.1%) | <0.001 |
| **Infectious diseases** | **A00 - B99** |  | **969 (7.1%)** | **681 (5.5%)** | **288 (24.5%)** | **<0.001** |
|  | A41.9 | Sepsis, unspecified organism | 238 (1.7%) | 160 (1.3%) | 78 (6.6%) | <0.001 |
|  | B34.8 | Other viral infections of unspecified site | 168 (1.2%) | 124 (1%) | 44 (3.7%) | <0.001 |
| **Genitourinary** | **N00 - N99** |  | **761 (5.6%)** | **560 (4.5%)** | **201 (17.1%)** | **<0.001** |
|  | N17.9 | Acute kidney failure, unspecified | 195 (1.4%) | 116 (0.9%) | 79 (6.7%) | <0.001 |
|  | N39.0 | Urinary tract infection, site not specified | 161 (1.2%) | 118 (0.9%) | 43 (3.7%) | <0.001 |
|  | N18.9 | Chronic kidney disease, unspecified | 155 (1.1%) | 128 (1%) | 27 (2.3%) | <0.001 |
| **Respiratory failure** | **J96, J80, J95.82, R09.2** |  | **716 (5.2%)** | **436 (3.5%)** | **280 (23.8%)** | **<0.001** |
|  | J96.00 | Acute respiratory failure, unspecified whether with hypoxia or hypercapnia | 279 (2%) | 150 (1.2%) | 129 (11%) | <0.001 |
|  | J96.01 | Acute respiratory failure with hypoxia | 228 (1.7%) | 166 (1.3%) | 62 (5.3%) | <0.001 |
| **Problems originating in perinatal period** | **P00 - P96** |  | **701 (5.1%)** | **427 (3.4%)** | **274 (23.3%)** | **<0.001** |
|  | P07.30 | Preterm newborn, unspecified weeks of gestation | 134 (1%) | 99 (0.8%) | 35 (3%) | <0.001 |
| **Musculo-skeletal diseases** | **M00 - M99** |  | **627 (4.6%)** | **468 (3.8%)** | **159 (13.5%)** | **<0.001** |
|  | M62.89 | Other specified disorders of muscle | 46 (0.3%) | 32 (0.3%) | 14 (1.2%) | <0.001 |
| **Developmental disorders** | **F01 - F99** |  | **604 (4.4%)** | **446 (3.6%)** | **158 (13.4%)** | **<0.001** |
|  | F88 | Other disorders of psychological development | 126 (0.9%) | 100 (0.8%) | 26 (2.2%) | <0.001 |
| **Ear and mastoid diseases** | **H60 – H59** |  | **511 (3.7%)** | **401 (3.2%)** | **110 (9.4%)** | **<0.001** |
|  | H54.7 | Unspecified visual loss | 36 (0.3%) | 28 (0.2%) | 8 (0.7%) | 0.01 |
| **Cyanotic heart diseases** | **Q24.9, Q26.2, Q21.3, Q22, Q20.3** |  | **451 (3.3%)** | **307 (2.5%)** | **144 (12.2%)** | **<0.001** |
|  | Q24.9 | Congenital malformation of heart, unspecified | 161 (1.2%) | 89 (0.7%) | 72 (6.1%) | <0.001 |
|  | Q21.3 | Tetralogy of Fallot | 157 (1.2%) | 112 (0.9%) | 45 (3.8%) | <0.001 |
| **Neoplasms** | **C00 - D49** |  | **402 (2.9%)** | **304 (2.4%)** | **98 (8.3%)** | **<0.001** |
|  | C91.00 | Acute lymphoblastic leukemia not having achieved remission | 84 (0.6%) | 62 (0.5%) | 22 (1.9%) | <0.001 |
| **Skin diseases** | **L00 - L99** |  | **359 (2.6%)** | **277 (2.2%)** | **82 (7%)** | **<0.001** |
|  | 0.9 | Dermatitis, unspecified | 37 (0.3%) | 8 (0.7%) | 45 (0.3%) | <0.001 |
| **External causes of morbidity** | **V00 - Y99** |  | **165 (1.2%)** | **159 (1.3%)** | **6 (0.5%)** | **0.017** |
|  | V89.2XXA | Person injured in unspecified motor-vehicle accident, traffic, initial encounter | 36 (0.3%) | 2 (0.2%) | 38 (0.3%) | <0.001 |

^*^*p*-values were computed using fisher exact test to test independence between MV outcome and presence of each diagnostic category/code.

**S1 Table 5.** **Prevalence of diagnostic categories across risk groups obtained on positive predictions from the entire dataset.**

| **Diagnostic category** | **ICD-10**  **Diagnostic code(s)** | **Prevalence across all positive predictions N(%)** | **Prevalence: N (%)** | | | | ***p*-value^*^ for trend** |
| --- | --- | --- | --- | --- | --- | --- | --- |
|  |  |  | **High risk group** | **Moderate risk group** | | **Low risk group** |  |
| Other unclassified diagnosis | R00 - R99 | 835 (72.9%) | 221 (72.2%) | 383 (72.5%) | | 231 (74%) | 0.611 |
| Health services (for non-disease encounters) | Z00 - Z99 | 773 (67.5%) | 239 (78.1%) | 353 (66.9%) | | 181 (58%) | <0.001 |
| Cardiovascular | I00 - I99, Q20 - Q28, R00, R01, R03 | 767 (66.9%) | 244 (79.7%) | 360 (68.2%) | | 163 (52.2%) | <0.001 |
| Respiratory | J00 - J99, Q30 - Q34, P22 - P28, R04 - R07, R09 | 626 (54.6%) | 171 (55.9%) | 316 (59.8%) | | 139 (44.6%) | 0.004 |
| Congenital diseases | Q00 - Q99 | 602 (52.5%) | 222 (72.5%) | 273 (51.7%) | | 107 (34.3%) | <0.001 |
| Congenital heart diseases | Q20 - Q28 | 461 (40.2%) | 187 (61.1%) | 211  (40%) | | 63 (20.2%) | <0.001 |
| Hematologic and immune disorders | D50 - D89 | 401 (35%) | 84 (27.5%) | 185  (35%) | | 132 (42.3%) | <0.001 |
| Metabolic disorders | E00 - E89 | 365 (31.8%) | 83 (27.1%) | 173 (32.8%) | | 109 (34.9%) | 0.038 |
| Gastro-intestinal | K00 - K95 | 309  (27%) | 78 (25.5%) | 136 (25.8%) | | 95 (30.4%) | 0.163 |
| Infectious diseases | A00 - B99 | 288 (25.1%) | 60 (19.6%) | 141 (26.7%) | | 87 (27.9%) | 0.018 |
| Neurologic disorders | G00 - G99 | 268 (23.4%) | 56 (18.3%) | 109 (20.6%) | | 103 (33%) | <0.001 |
| Problems originating in perinatal period | P00 - P96 | 247 (21.6%) | 93 (30.4%) | 133 (25.2%) | | 21 (6.7%) | <0.001 |
| Injury/trauma | S00 - T88 | 238 (20.8%) | 57 (18.6%) | 89 (16.9%) | | 92 (29.5%) | <0.001 |
| Genitourinary | N00 - N99 | 203 (17.7%) | 50 (16.3%) | 84 (15.9%) | | 69 (22.1%) | 0.059 |
| Respiratory failure | J96, J80, J95.82, R09.2 | 177 (15.4%) | 50 (16.3%) | 100 (18.9%) | | 27 (8.7%) | 0.008 |
| Musculo-skeletal diseases | M00 - M99 | 154 (13.4%) | 34 (11.1%) | 76 (14.4%) | | 44 (14.1%) | 0.278 |
| Cyanotic heart diseases | Q24.9, Q26.2, Q21.3, Q22, Q20.3 | 147 (12.8%) | 67 (21.9%) | 66 (12.5%) | | 14 (4.5%) | <0.001 |
| Developmental disorders | F01 - F99 | 137  (12%) | 29  (9.5%) | | 61 (11.6%) | 47 (15.1%) | 0.032 |
| Neoplasms | C00 - D49 | 132 (11.5%) | 18  (5.9%) | | 47 (8.9%) | 67 (21.5%) | <0.001 |
| Ear and mastoid diseases | H60 – H59 | 113  (9.9%) | 18  (5.9%) | | 43 (8.1%) | 52 (16.7%) | <0.001 |
| Skin diseases | L00 - L99 | 88  (7.7%) | 22  (7.2%) | | 33 (6.2%) | 33 (10.6%) | 0.111 |
| Sepsis |  | 37  (3.2%) | 7  (2.3%) | | 23 (4.4%) | 7  (2.2%) | 0.965 |
| External causes of morbidity | V00 - Y99 | 5  (0.4%) | 1  (0.3%) | | 0  (0%) | 4  (1.3%) | 0.07 |

^*^*p*-value was determined using Cochran-Armitage test for trend in proportion of each diagnostic category across the ordered risk groups.

**S1 Table 6. Comparison of test set performance metrics across model-B and its variants with dual threshold (model-B1) and waiting period (model-B2) based prediction schemes.**

| Performance metrics | Model-B: single threshold (default) | Model-B1:  dual threshold (second threshold T hours apart from first threshold) | | | Model-B2:  single threshold with waiting period of T hours | | |
| --- | --- | --- | --- | --- | --- | --- | --- |
|  |  | **T = 2 hours** | **T = 4 hours** | **T = 6 hours** | **T = 2 hours** | **T = 4 hours** | **T = 6 hours** |
| AUROC | 0.86 | 0.86 | 0.87 | 0.88 | 0.86 | 0.86 | 0.86 |
| AUPRC | 0.46 | 0.48 | 0.48 | 0.49 | 0.46 | 0.45 | 0.46 |
| Sensitivity (recall) | 0.43 | 0.37 | 0.44 | 0.42 | 0.40 | 0.40 | 0.42 |
| Specificity | 0.95 | 0.96 | 0.94 | 0.95 | 0.96 | 0.96 | 0.94 |
| Positive predictive value (precision) | 0.45 | 0.44 | 0.42 | 0.42 | 0.46 | 0.46 | 0.41 |
| Negative predictive value | 0.95 | 0.94 | 0.95 | 0.95 | 0.94 | 0.94 | 0.95 |
| F1 score | 0.44 | 0.40 | 0.43 | 0.42 | 0.43 | 0.43 | 0.42 |
| Early warning time^*^ (hours) | 40.25 [11.0 – 134.3] | 70.2 [23.3 – 153.6] | 58.2 [20.8 – 155.7] | 60.1 [25.0 – 159.6] | 69.2 [21.1 – 157.0] | 57.6 [21.6 – 183.1] | 59.7 [25.0 – 172.9] |

^*^Expressed as median [IQR]
AUROC: Area under receiver operating characteristic curve
AUPRC: area under precision-recall curve
